# Predictors of delayed care seeking for tuberculosis in southern India: an observational study

**DOI:** 10.1186/s12879-017-2629-9

**Published:** 2017-08-15

**Authors:** Sarah E. Van Ness, Ankit Chandra, Sonali Sarkar, Jane Pleskunas, Jerrold J. Ellner, Gautam Roy, Subitha Lakshminarayanan, Swaroop Sahu, C. Robert Horsburgh, Helen E. Jenkins, Natasha S. Hochberg

**Affiliations:** 1 Department of Biostatistics, Boston University, Crosstown Building, 801 Massachusetts Avenue, 3rd Floor, Boston, MA 02118 USA; 2 Department of Preventive and Social Medicine, JIPMER, Puducherry, India; 3 Boston Medical Center, Boston, MA USA; 4 Department of Medicine, Section of Infectious Diseases, Boston University School of Medicine, Boston, MA USA; 5 Department of Epidemiology, Boston University School of Public Health, Boston, MA USA

**Keywords:** Alcohol, Knowledge, Income, Cough

## Abstract

**Background:**

Reducing delay to accessing care is necessary to reduce the Tuberculosis (TB) burden in high incidence countries such as India. This study aimed to identify factors associated with delays in seeking care for TB in Southern India.

**Methods:**

We analyzed data from newly diagnosed, smear-positive, culture-confirmed, pulmonary TB patients in the Regional Prospective Observational Research for TB (RePORT) cohort in Puducherry and Tamil Nadu, India. Data were collected on demographic characteristics, symptom duration, and TB knowledge, among other factors. Delay was defined as cough ≥4 weeks before treatment initiation. Risky alcohol use was defined by the AUDIT-C score which incorporates information about regular alcohol use and binge drinking. TB knowledge was assessed by knowing transmission mode or potential curability.

**Results:**

Of 501 TB patients, 369 (73.7%) subjects delayed seeking care. In multivariable analysis, risky alcohol use was significantly associated with delay (aOR 2.20, 95% CI: 1.31, 3.68). Delay was less likely in lower versus higher income groups (<3000 versus >10,000 rupees/month, aOR 0.31, 95% CI: 0.12, 0.78). TB knowledge was not significantly associated with delay.

**Conclusions:**

Local TB programs should consider that risky alcohol users may delay seeking care for TB. Further studies will be needed to determine why patients with higher income delay in seeking care.

**Electronic supplementary material:**

The online version of this article (doi:10.1186/s12879-017-2629-9) contains supplementary material, which is available to authorized users.

## Background

In 2015 there were 2.8 million incident tuberculosis (TB) cases in India, comprising 27% of the 10.4 million global estimated TB cases [1, 2]. Reducing TB incidence will require a multi-pronged approach including efforts to decrease time to accessing care. Untreated, TB patients spread infection and increase their own mortality risk [3–9].

Although there have been several studies in India of predictors of delay in seeking care, results have been mixed and are likely setting-specific in such a large heterogeneous country [5, 10–16]. Previous studies have found that longer delay is associated with factors including income, substance abuse, location of first seeking care, and distance to health care facility among others [5, 10–16]. However other studies found no association with income and alcohol use [10, 11, 13, 16].

One potential component of delay is the disconnect between different healthcare systems. Patients can obtain healthcare through public and private clinics or a mix of both. The Revised National TB Control Programme (RNTCP) offers free TB diagnosis and treatment to patients in public clinics [17]. However, a recent study found that more than a quarter of prevalent TB patients are never evaluated at RNTCP clinics [18] and review of drug sales show that this number is likely even higher [1]. Patients often follow a circuitous route to diagnosis including self-medication and consulting private practitioners which often results in delayed diagnosis and treatment initiation [5, 11, 14–16]. A study of standardized patients found that only 21% of the cases were properly managed by private healthcare providers [17, 19]. More work is needed to understand factors associated with delay in the private and public sectors which include the time it takes patients to enter the health system and how long until they are started on TB treatment.

Previous studies have shown that hazardous alcohol use is prevalent in India [20–24]. The World Health Organization (WHO) estimated that 25% of the country’s men have consumed alcohol in the past year [24]. Other studies in India have found risky alcohol use in 40% of all males who consume alcohol [20, 22] and 52% of all male TB patients who consume alcohol [23]. Furthermore, in 2014, the WHO reported that alcohol use in India has increased in the last five years [25]. Alcohol use has been shown to be associated with poor TB treatment outcomes [23, 26] however, the degree of association between delays in seeking care and alcohol use has not been consistently demonstrated, possibly due to varying definitions of high alcohol use and regional differences in alcohol consumption [11–13, 27, 28]. Furthermore, while knowledge about TB has been identified as a reason for delay in qualitative studies [29, 30], to our knowledge, only one study in India has examined this association quantitatively [12]. We aimed to identify predictors of delay to treatment initiation at RNTCP clinics for new TB patients in Puducherry and Tamil Nadu to help inform public health interventions.

## Methods

### Study population and recruitment

This study is part of an ongoing observational household contact study in Puducherry and the Villapuram and Cuddalore districts of Tamil Nadu in southern India; the Regional Prospective Observational Research for TB (RePORT) study [31]. All new smear-positive patients are recruited from the 132 selected RNTCP designated microscopy centers (DMCs) and primary healthcare centers (PHCs) that are closest to Puducherry and see a large number of TB cases, out of a total of 305 centers. In brief, inclusion criteria include: Age ≥ 6 years; new TB diagnosis (category 1) with no known history of TB or TB treatment; smear-positive, culture-confirmed pulmonary TB; culture-negative cases are retrospectively excluded. Exclusion criteria include: having taken ≥3 doses of TB medication; known multi-drug resistance (MDR) infection (TB strain resistant to at least isoniazid and rifampicin) or contact with MDR case; not planning to continue treatment.

### Data collection and study definitions

Study staff administered a questionnaire addressing demographic and clinical characteristics including age, gender, marital status, religion, caste, education level, monthly household income, alcohol use, smoking status, and knowledge of TB (Additional file 1). Most interviews were conducted in the clinic, although some were conducted in the subject’s home. Paper questionnaires were scanned and transferred to Boston University with Verity TeleForm Information Capture System software V10.8 (Sunnyvale, CA, USA), and read into a Microsoft Access (Seattle, WA, USA) database. Any errors were reviewed and corrected by the study team in India. Risky alcohol use was determined by AUDIT-C, a validated screening tool designed to identify individuals with hazardous drinking behavior by frequency and volume of alcohol consumption and binge-drinking [32, 33]. For example, a subject could be considered a risky drinker if they reported drinking alcohol: 2–3 times per week and having ≥3 drinks on a typical day; or 2–4 times a month and having ≥6 drinks in one sitting at least monthly. Subjects were shown standardized illustrations of the amount of alcohol that constitutes one drink.

To address knowledge of TB, participants were asked whether TB is curable and how it is transmitted. Individuals were provided with a list of options (coughing, sharing utensils, sharing clothes, smoking, touch, food, sexual contact, and mosquito bites) and they could choose as many answers as they believed appropriate. Correct knowledge of transmission was defined as choosing coughing regardless of whether other answers were chosen.

Individuals were asked about presence and duration of TB symptoms. Delay to accessing care was defined as duration from symptom onset until study enrollment (i.e. had received at most 3 doses of treatment). Symptom onset was determined by cough duration dichotomized by the median; those with a cough ≥4 weeks were defined as delayed in accessing care. The 4 week cutoff was chosen because median cough duration and cough duration of one month have both been used in previous studies of delay in accessing care for TB [4, 5, 34].

Patients were also asked where they first sought care; answers were dichotomized into government and private facilities based on whether the patient had to pay for treatment. Government facilities included government hospitals, PHCs, and municipal corporation hospitals. Private facilities included private allopathic clinics, non-allopathic clinics, medicine shops, and any other site. Although medical colleges could fit into either category, after discussion with study staff, these were allocated into private facilities.

### Data analysis

Data were analyzed using SAS 9.4. Continuous variables were dichotomized by their medians. Both univariate and multivariable analyses were performed using logistic regression with delay as the outcome variable. Variables with *p*-values ≤0.2 in the univariate regression, as well as age and sex, were included in the multivariable model. Location of first care was also included in the model to account for the fact that delay could differ for those who visited other providers before RNTCP. Backward selection was considered but all variables with *p*-values ≤0.2 were of interest so none were removed. For those variables associated with delay in the multivariable model, logistic regression was used to assess associations with other predictors. We also examined interactions between variables that were associated with delay in the multivariable model.

## Results

### Socio-demographic characteristics

Overall, 656 TB patients who met study inclusion criteria were reported by the PHCs and DMCs from May 1, 2014 through July 27, 2016; of these, 638 (97%) were able to be located and were contacted regarding study enrollment. Of the 638, 514 (81%) consented to participate, and 501 were included in this analysis (3 were missing information on cough and 10 were missing Mtb culture data or other key variables).The subjects included 366 (73.1%) from Puducherry and 135 (26.9%) from Cuddalore and Villupuram in Tamil Nadu (Table 1). The mean age was 43.8 years (range: 14–81); 385 (76.8%) were men and 204 (40.7%) were risky alcohol users. The majority (352; 75.4%) had monthly household incomes between 3000 and 10,000 rupees. Most subjects (469; 93.6%) knew that TB is curable and 382 (76.2%) knew that it is transmitted by coughing. Many subjects (304; 61%) first sought care somewhere other than RNTCP clinics, including private clinics (253; 50.8%), pharmacies (24; 4.8%), medical colleges (21, 4.2%) and non-allopathic clinics (2; 0.4%).

**Table 1:** Socio-demographic characteristics of included participants

Characteristic		n(%)
Age	≥ 45 years	266 (53.3%)
	> 45 years	233 (46.7%)
Sex	Male	385 (76.8%)
	Female	116 (23.2%)
BMI	Underweight (BMI < 18.5)	306 (61.7%)
	Normal (18.5 ≤ BMI ≤ 25)	168 (33.9%)
	Overweight (BMI > 25)	22 (4.4%)
Employment	Employed	400 (80.0%)
	Unemployed	64 (12.8%)
	Student	36 (7.2%)
Household Income per month	< 3000 rupees	69 (14.2%)
	3000–5000 rupees	180 (37.0%)
	5001–10,000 rupees	172 (35.4%)
	> 10,000 rupees	65 (13.4%)
Household Size	≥ 4 people	269 (53.8%)
	< 4 people	231 (46.2%)
Location of First Care^a^	Private	304 (61.0%)
	Government	194 (39.0%)
Marital Status	Married/Living together	342 (68.3%)
	Not married	159 (31.7%)
Caste^b^	Other backward caste	356 (72.7%)
	Scheduled caste	134 (27.3%)
Municipality	Puducherry	366 (73.1%)
	Tamil Nadu	135 (26.9%)
Religion	Hinduism	447 (89.4%)
	Other	53 (10.6%)
Risky Alcohol Use^c^	No	297 (59.3%)
	Yes	204 (40.7%)
Smoking Status	Never	257 (51.3%)
	Yes (former/current)	244 (48.7%)
Mother’s Education	0 Years	372 (77.2%)
	> 0 Years	110 (22.8%)
Years of School	≤ 7 years	252 (50.6%)
	> 7 years	246 (49.4%)
Knowledge that TB is transmitted by cough	Yes	382 (76.2%)
	No	119 (23.8%)
Knowledge that TB is curable	Yes	469 (93.6%)
	No	32 (6.4%)

^a^Private facilities include pharmacies, private allopathic clinics, medical college hospitals, and non-allopathic clinics. Government facilities included government hospitals, PHCs, and municipal corporation hospitals

^b^Scheduled castes and tribes are among the most disadvantaged socio-economic groups in India. These groups have been designated by the Government of India to receive special programming and legislation to promote empowerment and development

^c^Risky alcohol use was assessed using the AUDIT-C score, which incorporates information based on habitual frequency and volume of alcohol use as well as binge drinking tendencies

### Delays in care and factors associated with delay

The mean cough duration was 3.59 weeks (standard deviation: 1.14) and median 4 weeks (range: 1–12); the majority of subjects (71.9%) reported a cough duration of exactly 4 weeks. Overall, 369 (73.7%) delayed seeking care (cough duration of ≥4 weeks).

The multivariable model included 480 individuals; 21 were excluded due to missing data for one or more of the covariates. Risky alcohol use was associated with delay (adjusted odds ratio [aOR] 2.20; 95% confidence interval [CI]: 1.31, 3.68), after controlling for other factors (Table 2). Household income was also independently associated with delay. Subjects from households earning <3000 rupees were less likely to delay than those earning >10,000 rupees/month (aOR 0.31, 95% CI: 0.12–0.78; Table 2). Neither knowledge of transmission nor cure was associated with delay in multivariable models. Location where individuals first sought care (public vs. private facility) was not significantly associated with delay (aOR 1.25; 95% CI: 0.80, 1.96). However, the location of first care was associated with household income (*p* = 0.002); 76.9% of subjects in the highest income group visited a private facility first compared to 47.8% in the lowest income group.

**Table 2:** Univariate and multivariable analysis of risk factors for delay in engagement in care

				Univariate		Multivariable	
Characteristic		Cough <4 weeks n(%)	Cough ≥4 weeks n(%)	OR (95% CI)	*p*-value*	OR (95% CI)	*p*-value**
Age	≤ 45 years	78 (29.3%)	188 (70.7%)	Reference		Reference	
	> 45 years	54 (23.2%)	179 (76.8%)	1.38 (0.92, 2.06)	0.12	1.18 (0.76, 1.83)	0.46
Sex	Male	96 (24.9%)	289 (75.1%)	Reference		Reference	
	Female	36 (31.0%)	80 (69.0%)	0.74 (0.47, 1.16)	0.19	1.22 (0.68, 2.21)	0.51
BMI	Underweight (BMI < 18.5)	43 (25.6%)	125 (74.4%)	Reference			
	Normal (18.5 ≤ BMI ≤ 25)	7 (31.8%)	15 (68.2%)	0.74 (0.28, 1.93)	0.53		
	Overweight (BMI > 25)	81 (26.5%)	225 (73.5%)	0.96 (0.62, 1.47)	0.84		
Employment	Employed	106 (26.5%)	294 (73.5%)	Reference			
	Student	8 (22.2%)	28 (77.8%)	1.26 (0.56, 2.86)	0.58		
	Unemployed	18 (28.1%)	46 (71.9%)	0.92 (0.51, 1.66)	0.79		
Household Income per month	> 10,000 rupees	10 (15.4%)	55 (84.6%)	Reference		Reference	
	5001–10,000 rupees	42 (24.4%)	130 (75.6%)	0.56 (0.26, 1.20)	0.14	0.47 (0.22, 1.02)	0.057
	3000–5000 rupees	61 (33.9%)	119 (66.1%)	0.35 (0.17, 0.74)	0.006	0.27 (0.12, 0.57)	<0.001
	< 3000 rupees	17 (24.6%)	52 (75.4%)	0.56 (0.23, 1.33)	0.19	0.31 (0.12, 0.78)	0.014
Household Size	< 4 people	55 (23.8%)	176 (76.2%)	Reference			
	≥ 4 people	77 (28.6%)	192 (71.4%)	0.78 (0.52, 1.16)	0.22		
Location of First Care^a^	Private	85 (28.0%)	219 (72.0%)	Reference		Reference	
	Government	45 (23.2%)	149 (76.8%)	1.29 (0.85, 1.95)	0.24	1.25 (0.80, 1.96)	0.33
Marital Status	Married/Living together	87 (25.4%)	255 (74.6%)	Reference			
	Not married	45 (28.3%)	114 (71.7%)	0.86 (0.57, 1.32)	0.50		
Caste^b^	Other backward caste	93 (26.1%)	263 (73.9%)	Reference			
	Scheduled caste	35 (26.1%)	99 (73.9%)	1.00 (0.64, 1.57)	>0.99		
Municipality	Puducherry	100 (27.3%)	266 (72.7%)	Reference			
	Tamil Nadu	32 (23.7%)	103 (76.3%)	1.21 (0.77, 1.91)	0.42		
Religion	Hinduism	113 (25.3%)	334 (74.7%)	Reference		Reference	
	Other	19 (35.8%)	34 (64.2%)	0.61 (0.33, 1.10)	0.10	0.68 (0.36, 1.28)	0.23
Risky Alcohol Use^c^	No	94 (31.6%)	203 (68.4%)	Reference		Reference	
	Yes	38 (18.6%)	166 (81.4%)	2.02 (1.32, 3.11)	0.001	2.20 (1.31, 3.68)	0.003
Smoking Status	Never	76 (29.6%)	181 (70.4%)	Reference		Reference	
	Yes (former/current)	56 (23.0%)	188 (77.0%)	1.41 (0.94, 2.10)	0.093	1.15 (0.69, 1.94)	0.59
Mother’s Education	Some	28 (25.5%)	82 (74.5%)	Reference			
	None	100 (26.9%)	272 (73.1%)	0.93 (0.57, 1.51)	0.77		
Years of School	> 7 years	62 (25.2%)	184 (74.8%)	Reference			
	≤ 7 years	70 (27.8%)	182 (72.2%)	0.88 (0.59, 1.31)	0.52		
Knowledge that TB is transmitted by cough	Yes	106 (27.7%)	276 (72.3%)	Reference		Reference	
	No	26 (21.8%)	93 (78.2%)	1.37 (0.84, 2.24)	0.20	1.09 (0.62, 1.92)	0.77
Knowledge that TB is curable	Yes	128 (27.3%)	341 (72.7%)	Reference		Reference	
	No	4 (12.5%)	28 (87.5%)	2.63 (0.90, 7.64)	0.076	2.04 (0.66, 6.33)	0.22

*p-value calculated using logistic regression

**All covariates with univariate *p*-values less than or equal to 0.2 plus age, sex, and location of first care were included in the multivariable model

^a^Private facilities include pharmacies, private allopathic clinics, medical college hospitals, and non-allopathic clinics. Government facilities included government hospitals, PHCs, and municipal corporation hospitals

^b^Scheduled castes and tribes are among the most disadvantaged socio-economic groups in India. These groups have been designated by the Government of India to receive special programming and legislation to promote empowerment and development

^c^Risky alcohol use was assessed using the AUDIT-C score, which incorporates information based on habitual frequency and volume of alcohol use as well as binge drinking tendencies

The interaction term between income and risky alcohol use was not statistically significant (*p* = 0.064) but did indicate that the trend for people with higher income to be more likely to experience delay was stronger among people without risky alcohol use. Since some medical colleges charge for services and some do not, the analysis was also performed with medical colleges classified as public; this change had no effect on the conclusions.

### Characteristics of risky alcohol users

Of the 204 risky drinkers, 203 (99.5%) were male (Table 3). Risky alcohol users were more likely to be older (OR 1.58; 95% CI: 1.10, 2.27), employed (OR 4.00; 95% CI: 2.07, 7.72), from low income households (<3000 versus >10,000 rupees/month, OR 5.87, 95% CI: 2.75, 12.51), and to visit a government facility first (OR 1.74; 95% CI: 1.21, 2.51). In addition, risky alcohol users were more likely to not know that TB was curable (OR 2.98; 95% CI: 1.41, 6.33) or that it was transmitted by cough (OR 2.90; 95% CI: 1.90, 4.43).

**Table 3:** Characteristics of risky alcohol users

		Risky alcohol use^a^			
Characteristic		No n(%)	Yes n(%)	OR (95% CI)	*p*-value^†^
Age	≤ 45 years	171 (58.0%)	95 (46.6%)	Reference	
	> 45 years	124 (42.0%)	109 (53.4%)	1.58 (1.10, 2.27)	0.012
Sex	Female	115 (38.7%)	1 (0.5%)	Reference	
	Male	182 (61.3%)	203 (99.5%)	128.21 (17.73, 926.92)	<0.001
BMI	Underweight (BMI < 18.5)	107 (36.3%)	61 (30.3%)	Reference	
	Normal (18.5 ≤ BMI ≤ 25)	19 (6.4%)	3 (1.5%)	0.28 (0.08, 0.97)	0.045
	Overweight (BMI > 25)	169 (57.3%)	137 (68.2%)	1.42 (0.97, 2.09)	0.074
Employment	Unemployed	52 (17.6%)	12 (5.9%)	Reference	
	Employed	208 (70.3%)	192 (94.1%)	4.00 (2.07, 7.72)	<0.001
	Student	36 (12.2%)	0 (0.0%)	--^b^	--
Household Income per month	> 10,000 rupees	50 (17.5%)	15 (7.5%)	Reference	
	5001–10,000 rupees	103 (36.1%)	69 (34.3%)	2.23 (1.16, 4.29)	0.016
	3000–5000 rupees	107 (37.5%)	73 (36.3%)	2.27 (1.19, 4.35)	0.013
	< 3000 rupees	25 (8.8%)	44 (21.9%)	5.87 (2.75, 12.51)	<0.001
Household Size	< 4 people	145 (49.0%)	86 (42.2%)	Reference	
	≥ 4 people	151 (51.0%)	118 (57.8%)	1.32 (0.92, 1.89)	0.13
Location of First Care^c^	Private	196 (66.4%)	108 (53.2%)	Reference	
	Government	99 (33.6%)	95 (46.8%)	1.74 (1.21, 2.51)	0.003
Marital Status	Not married	128 (43.1%)	31 (15.2%)	Reference	
	Married/Living together	169 (56.9%)	173 (84.8%)	4.23 (2.71, 6.60)	<0.0001
Caste^d^	Other backward caste	217 (74.6%)	139 (69.8%)	Reference	
	Scheduled caste	74 (25.4%)	60 (30.2%)	1.27 (0.85, 1.89)	0.25
Municipality	Puducherry	224 (75.4%)	142 (69.6%)	Reference	
	Tamil Nadu	73 (24.6%)	62 (30.4%)	1.34 (0.90, 2.00)	0.15
Religion	Hinduism	258 (87.2%)	189 (92.6%)	Reference	
	Other	38 (12.8%)	15 (7.4%)	0.54 (0.29, 1.01)	0.053
Smoking Status	Never	205 (69.0%)	52 (25.5%)	Reference	
	Yes (former/current)	92 (31.0%)	152 (74.5%)	6.51 (4.37, 9.71)	<0.001
Mother’s Education	Some	86 (29.8%)	24 (12.4%)	Reference	
	None	203 (70.2%)	169 (87.6%)	2.98 (1.82, 4.90)	<0.001
Years of School	> 7 years	175 (58.9%)	71 (35.3%)	Reference	
	≤ 7 years	122 (41.1%)	130 (64.7%)	2.63 (1.81, 3.80)	<0.001
Knowledge that TB is transmitted by cough	Yes	250 (84.2%)	132 (64.7%)	Reference	
	No	47 (15.8%)	72 (35.3%)	2.90 (1.90, 4.43)	<0.001
Knowledge that TB is curable	Yes	286 (96.3%)	183 (89.7%)	Reference	
	No	11 (3.7%)	21 (10.3%)	2.98 (1.41, 6.33)	0.004

^†^ *p*-value calculated using logistic regression

^a^Risky alcohol use was assessed using the AUDIT-C score, which incorporates information based on habitual frequency and volume of alcohol use as well as binge drinking tendencies

^b^OR cannot be calculated because there are no student risky alcohol users

^c^Private facilities include pharmacies, private allopathic clinics, medical college hospitals, and non-allopathic clinics. Government facilities included government hospitals, PHCs, and municipal corporation hospitals

^d^Scheduled castes and tribes are among the most disadvantaged socio-economic groups in India. These groups have been designated by the Government of India to receive special programming and legislation to promote empowerment and development

## Discussion

This study of newly diagnosed pulmonary TB patients in Puducherry and Tamil Nadu, India found that risky alcohol users and those with higher income levels were more likely to delay seeking care at RNTCP clinics. Identifying and addressing these predictors may improve the time to treatment initiation and reduce transmission.

Previous studies of the association between alcohol use and engagement in care in India report mixed results. Studies of new pulmonary TB patients in Wardha District in western India (*n* = 53) and Tamil Nadu in the south (*n* = 531) found that alcoholism (categorized into “never”, “sometimes”, and “regular” consumption, and habitual alcohol consumption respectively) was associated with delay [11, 27]. Other studies in Mandi District in northern India (*n* = 234) and Tamil Nadu (*n* = 601) found no association; the former categorized alcohol use as daily use, and the latter study did not differentiate between casual and risky drinkers [12, 13]. Importantly, these studies used several less-standardized measures of alcohol use which could have masked the association with delay. The AUDIT-C used in our study is a validated measure of risky alcohol use, providing a standardized definition that has been applied in a variety of settings [32, 33]. Regional differences in alcohol use also may affect its role in delaying access to care [28, 35].

Because 40.7% of TB patients were risky alcohol users in this cohort, these results demonstrate a need to target risky drinkers in Puducherry and Tamil Nadu to encourage earlier TB diagnosis. We found risky alcohol use was associated with being male, married, having lower income (but being employed), and having less education – all characteristics that have been found to be associated with alcohol use in other studies [20, 28, 35]. Understanding the characteristics of risky alcohol users in this population may help public health providers design interventions to achieve this goal.

In our study, higher income was associated with delay in seeking care. This is in contrast to other studies in India that found that lower income individuals were more likely to delay [12, 15]. This difference could be due to income definitions and analysis techniques. We defined income as household income and use multivariable analysis, while one study used per-capita income and also did not control for any confounders [15] Furthermore, a study in northern India that found low income was associated with delay used a dichotomized household income variable while our study classified income into four levels [12].

The association between higher income and delay could also be related to the type of clinic where a patient first sought care. Previous studies have shown that patients visiting a government facility first are likely to have longer “patient delay” (onset of symptoms to first seeking health care) while visiting a private facility first is associated with longer “health system delay” (time from first seeking care until diagnosis or treatment initiation) [5, 11–14, 34]. Because our study assessed total delay (patient and health system delays combined), we may not have been able to detect an association between location of first care and delay. Higher income participants were more likely than lower income participants to visit a private facility first, which could have led to longer health system delay thus increasing their total delay. The association between higher income and delay remained, however, when controlling for location of first care (i.e., even when controlling for whether a patient first went to a private clinic, higher income individuals had longer delay).

There could be other facets of the relationship between health-seeking behavior, delay, and income that our questionnaires did not capture – such as the number of facilities visited – which could contribute to longer delays. Higher income could also be associated with delay if these individuals do not suspect that they have TB or, because they perceive that TB is a disease of poverty [36, 37]. Higher income individuals may also delay seeking care at public facilities because they fear the stigma associated with TB which has been reported to affect health-seeking behavior [38–40]. The relationship between higher income, care at private clinics, stigma, and delay should be investigated further to understand the connections between these factors and how best to encourage timely TB diagnoses.

Knowledge of TB transmission in India was found to be 55.5% in a country-wide survey and 65% in Puducherry [41, 42]. Our study did not find a significant association between delay and knowledge about TB, defined by curability or mode of transmission. This finding was surprising, as in previous studies patients cited poor knowledge as a reason for delayed care [11, 29, 30]. Furthermore, improved TB knowledge seems to play a role in engagement in care as following an educational intervention in Delhi, the number of individuals self-reporting with TB symptoms to a treatment center increased [43]. It is possible that we did not find an association between knowledge and delay because we only assessed knowledge of transmission and cure; some studies used numerous questions to create a knowledge score, allowing for finer categorization of knowledge levels [3, 12]. It also could be that in this area, delayed care is dominated by other factors, notably alcohol use or that patients’ knowledge of TB does not affect engagement in care because people seek care for symptoms not because they suspect they have a certain disease. We found that risky alcohol use was associated with poor TB knowledge suggesting that educational programs may be warranted for this group.

Our study had the following limitations. It is difficult to correctly ascertain household income due to inaccurate recall, which could introduce bias into the results about income. Similarly, the use of cough duration to quantify delay hinges upon a subject’s ability to recall symptom duration and there is no standard threshold that indicates delay, which could lead to ascertainment bias. However because there are no other well-defined measures of delay, cough duration with a cutoff at the median or at one month has been used by numerous other studies [4, 5, 34]. The median cough duration for our sample was four weeks therefore defining delay by the median fulfilled both of the common practices. We attempted to minimize inaccurate recall by interviewing subjects within one week of treatment initiation. Furthermore, although we asked participants where they first sought care, we were not able to ascertain the time spent getting evaluated at other clinics. Ultimately, we were still able to quantify the clinically relevant delay between symptom onset and treatment initiation, because the study excludes any individuals who previously received ≥3 doses of treatment (including at another clinic).

Our analysis demonstrated that public health measures to hasten engagement in care in Puducherry and Tamil Nadu should focus on risky drinkers. As of now, RNTCP training materials recognize the need to screen TB patients for alcohol use and encourage abstaining; we suggest targeting risky alcohol users, including those in alcohol treatment programs to enhance TB screening [44]. Additionally, higher income individuals delayed seeking care and studies are needed to determine the reasons for this delay, as it is not solely due to seeking care in private clinics. Social programs may need to target higher income groups with education and/or stigma-reduction campaigns to improve their engagement in care.

## Conclusion

As TB prevalence continues to rise in India, it is increasingly important to reduce the time that TB patients are infectious. In order to achieve this goal, the factors that contribute to delay in accessing care need to be thoroughly understood so that populations at risk for delay can be targeted. As stated by the WHO, a key component in eradicating TB worldwide will be the integration of TB control programs with other public health programs, such as for alcoholics in Southern India [2].

## Additional file

Additional file 1: The questionnaire that was used for data collection in this study. (PDF 139 kb)

## Abbreviations

aOR: Adjusted odds ratio
CI: Confidence interval
DMCs: Designated microscopy centers
MDR: Multi-drug resistance
PHCs: Primary healthcare centers
RePORT: Regional prospective observational research for TB
RNTCP: The revised national TB control programme
TB: Tuberculosis
WHO: World Health Organization

## References

[1] Arinaminpathy N, Batra D, Khaparde S, Vualnam T, Maheshwari N, Sharma L (2016). The number of privately treated tuberculosis cases in India : an estimation from drug sales data. Lancet Infect Dis.

[2] WHO. Global Tuberculosis Report 2016. 2016. http://www.who.int/tb/publications/global_report/en/. Accessed 11 Mar 2017.

[3] World Health Organization. Diagnostic and treatment delay in tuberculosis. 2006. http://applications.emro.who.int/dsaf/dsa710.pdf. Accessed 11 Mar 2017.

[4] Storla D, Yimer S, Bjune G (2008). A systematic review of delay in the diagnosis and treatment of tuberculosis. BMC Public Health.

[5] Sreeramareddy CT, Qin ZZ, Satyanarayana S, Subbaraman R, Pai M (2014). Delays in diagnosis and treatment of pulmonary tuberculosis in India: a systematic review. Int J Tuberc Lung Dis..

[6] Dye C, Williams BG (2010). The population dynamics and control of tuberculosis. Science.

[7] Madebo T, Lindtjorn B (1999). Delay in treatment of pulmonary tuberculosis: an analysis of symptom duration among Ethiopian patients. MedGenMed.

[8] Gebreegziabher SB, Bjune GA, Yimer SA (2016). Total delay is associated with unfavorable treatment outcome among pulmonary tuberculosis patients in west Gojjam zone, Northwest Ethiopia: a prospective cohort study. PLoS One.

[9] Toman K. Toman’s tuberculosis. WHO Library Cataloguing-in-Publication Data; 2004.

[10] Ananthakrishnan R, Jeyaraju AR, Palani G, Sathiyasekaran BWC (2012). Care seeking behavior of the TB patients who were registered in an urban government tuberculosis control in Chennai, Tamilnadu, India. J Clin Diagnostic Res.

[11] Rajeswari R, Chandrasekaran V, Suhadev M, Sivasubramaniam S, Sudha G, Renu G (2002). Factors associated with patient and health system delays in the diagnosis of tuberculosis in South India. Int J Tuberc Lung Dis..

[12] Thakur R, Murhekar M (2013). Delay in diagnosis and treatment among TB patients registered under RNTCP Mandi, Himachal, Pradesh. India Indian J Tuberc.

[13] Selvam JM, Wares F, Perumal M, Gopi PG, Sudha G, Chandrasekaran V (2007). Health-seeking behaviour of new smear-positive TB patients under a DOTS programme in Tamil Nadu, India, 2003. Int J Tuberc Lung Dis.

[14] Tamhane A, Ambe G, Vermund SH, Kohler CL, Karande A, Sathiakumar N (2012). Pulmonary tuberculosis in Mumbai, India: factors responsible for patient and treatment delays. Int J Prev Med.

[15] Kelkar-Khambete A, Kielmann K, Pawar S, Porter J, Inamdar V, Datye A (2008). India’s revised National Tuberculosis Control Programme: looking beyond detection and cure. Int J Tuberc Lung Dis..

[16] Sudha G, Nirupa C, Rajasakthivel M, Sivasusbramanian S, Sundaram V, Bhatt S (2003). Factors influencing the care-seeking behaviour of chest symptomatics: a community-based study involving rural and urban population in Tamil Nadu. South India Trop Med Int Heal.

[17] Central TB Division. 2015, TB India. New Delhi: Directorate general of health services, Ministry of Health and Family Welfare; 2015.

[18] Subbaraman R, Nathavitharana RR, Satyanarayana S, Pai M, Thomas BE, Chadha VK, et al. The Tuberculosis Cascade of Care in India’s Public Sector: A Systematic Review and Meta-analysis. PLOS Med. 2016;13. 10.1371/journal.pmed.1002149 PMC5079571 27780217

[19] Das J, Kwan A, Daniels B, Satyanarayana S, Subbaraman R, Bergkvist S (2015). Use of standardised patients to assess quality of tuberculosis care: a pilot, cross-sectional study. Lancet Infect Dis.

[20] John A, Barman A, Bal D, Chandy G, Samuel J, Thokchom M (2009). Hazardous alcohol use in rural southern India: nature, prevalence and risk factors. Natl Med J India.

[21] Pillai A, Nayak MB, Greenfield TK, Bond JC, Nadkarni A, Patel V (2013). Patterns of alcohol use, their correlates, and impact in male drinkers: a population-based survey from Goa, India. Soc Psychiatry Psychiatr Epidemiol.

[22] Rathod SD, Nadkarni A, Bhana A, Shidhaye R (2015). Epidemiological features of alcohol use in rural India: a population-based cross-sectional study. BMJ Open.

[23] Suhadev M, Thomas BE, Raja Sakthivel M, Murugesan P, Chandrasekaran V, Charles N, et al. Alcohol use disorders (AUD) among tuberculosis patients: a study from Chennai. South India PLoS One. 2011;6. 10.1371/journal.pone.0019485 PMC3096635 21611189

[24] World Health Organization. Global status report on alcohol and health. WHO country profile India; 2004. http://wwwwhoint/substance_abuse/publications/global_alcohol_report/profiles/indpdf?ua=1. Accessed 12 Mar 2017.

[25] World Health Organization. Global status report on alcohol and health. 2014. http://www.who.int/substance_abuse/publications/global_alcohol_report/en/. Accessed 11 Mar 2017.

[26] Lonnroth K, Williams BG, Stadlin S, Jaramillo E, Dye C (2008). Alcohol use as a risk factor for tuberculosis - a systematic review. BMC Public Health.

[27] Bawankule S, Zahiruddun QS, Gaidhane A, Khatib N (2010). Delay in DOTS for new pulmonary tuberculosis patient from rural area of Wardha District. India Online J Heal Allied Sci.

[28] Neufeld KJ, Peters DH, Rani M, Bonu S, Brooner RK (2005). Regular use of alcohol and tobacco in India and its association with age, gender, and poverty. Drug Alcohol Depend.

[29] Shiotani R, Hennink M (1692). Socio-cultural influences on adherence to tuberculosis treatment in rural India. Glob Public Health.

[30] McArthur E, Bali S, Khan AA (2016). Socio-cultural and knowledge-based barriers to tuberculosis diagnosis for women in Bhopal, India. Indian J Community Med.

[31] Hamilton CD, Swaminathan S, Christopher DJ, Ellner J, Gupta A, Sterling TR (2015). RePORT international: advancing tuberculosis biomarker research through global collaboration. Clin Infect Dis.

[32] Bradley KA, Bush KR, Epler AJ, Dobie DJ, Davis TM, Sporleder JL (2003). Two brief alcohol-screening tests from the alcohol use disorders identification test (AUDIT): validation in a female veterans affairs patient population. Arch Intern Med.

[33] Bush K, Kivlahan DR, McDonell MB, Fihn SD, Bradley KA (1998). The AUDIT alcohol consumption questions (AUDIT-C): an effective brief screening test for problem drinking. Ambulatory care quality improvement project (ACQUIP). Alcohol use disorders identification test. Arch Intern Med.

[34] Cai J, Wang X, Ma A, Wang Q, Han X, Li Y (2015). Factors associated with patient and provider delays for tuberculosis diagnosis and treatment in Asia: a systematic review and meta-analysis. PLoS One.

[35] Kim S, Rifkin S, John SM, Jacob KS (2013). Nature, prevalence and risk factors of alcohol use in an urban slum of southern India. Natl Med J India.

[36] Lönnroth K, Jaramillo E, Williams BG, Dye C, Raviglione M (2009). Drivers of tuberculosis epidemics: the role of risk factors and social determinants. Soc Sci Med.

[37] World Health Organization. A human rights approach to TB Stop TB Guidelines for social moblization. 2001. http://apps.who.int/iris/handle/10665/66701. Accessed 11 Mar 2017.

[38] Chang SH, Cataldo JK (2014). A systematic review of global cultural variations in knowledge, attitudes and health responses to tuberculosis stigma. Int J Tuberc Lung Dis..

[39] Courtwright A, Turner AN (2010). Tuberculosis and stigmatization: patways and interventions. Public Health Rep.

[40] Somma D, Thomas BE, Karim F, Kemp J, Arias N, Auer C (2008). Gender and socio-cultural determinants of TB-related stigma in Bangladesh, India, Malawi and Colombia. Int J Tubercu Lung Dis.

[41] Sreeramareddy CT, Harsha Kumar HN, Arokiasamy JT (2013). Prevalence of self-reported tuberculosis, knowledge about tuberculosis transmission and its determinants among adults in India: results from a nation-wide cross-sectional household survey. BMC Infect Dis.

[42] Chinnakali P, Ramakrishnan J, Vasudevan K, Gurumurthy J, Upadhyay RP, Panigrahi KC (2013). Level of awareness about tuberculosis in urban slums: implications for advocacy and communication strategy planning in the national program. Lung India.

[43] Sharma N, Taneja DK, Pagare D, Saha R, Vashist RP, Ingle GK (2005). The impact of an IEC campaign on tuberculosis awareness and health seeking behaviour in Delhi, India. Int J Tuberc Lung Dis..

[44] Goverment of India Central Tuberculosis Division. Managing the RNTCP in your area: A Training course (modules 1–4). 2014. http://www.tbcindia.nic.in/index1.php?lang=1&level=3&sublinkid=4262&lid=2906. Accessed 11 Mar 2017.

